# Carbon Dots-Enhanced Soy Protein Isolate/Polyvinyl Alcohol Composite Film for Active Preservation of Oxidation-Sensitive Foods

**DOI:** 10.3390/antiox14060669

**Published:** 2025-05-31

**Authors:** Linlin Zhao, Huinan Jiang, Zhengxuan Han, Wenqin Gu, Bimal Chitrakar, Xiangren Meng

**Affiliations:** 1 College of Tourism and Culinary Science, Yangzhou University, Yangzhou 225127, China; 008113@yzu.edu.cn (L.Z.); mz120232117@stu.yzu.edu.cn (H.J.); 212402502@stu.yzu.edu.cn (Z.H.); mx120241311@stu.yzu.edu.cn (W.G.); 2 Key Laboratory of Chinese Cuisine Intangible Cultural Heritage Technology Inheritance, Ministry of Culture and Tourism, Yangzhou 225127, China; 3 College of Food Science and Engineering, Yangzhou University, Yangzhou 225127, China; 4 College of Food Science and Technology, Hebei Agricultural University, Baoding 071001, China; bimal@hebau.edu.cn; 5 Chinese Cuisine Promotion and Research Base, Yangzhou 225127, China

**Keywords:** active packaging, carbon dots, antibacterial property, antioxidant activity

## Abstract

Carbon dots perform a variety of functions when they are incorporated into active films synthesized from soy protein isolate/polyvinyl alcohol. The study examined the impact of varying concentrations of carbon dots on the structural, bioactive, and physicochemical properties of composite films. The addition of carbon dots improved the UV-blocking rate (up to 99.64%) with a higher water-barrier property of the films. The films with carbon dot-enhanced antioxidant activities (ABTS was 99.71%, and DPPH was 51.44%) exhibited strong antibacterial activities against *Escherichia coli* and *Staphylococcus aureus*. In a hydrophilic environment, the high release rate of carbon dots was found to enhance the biological activity of films. The application of 0.8% composite film resulted in significant shelf-life extension for fresh jujubes, meatballs, and soybean oil. These results demonstrated the feasibility of composite films as an active packaging material.

## 1. Introduction

It is crucial to select appropriate packing materials to extend the freshness and shelf life of oxidation-sensitive foods, thereby reducing loss and waste [1,2]. Traditional food packaging materials, including polypropylene, polyethylene terephthalate, polystyrene, etc., cannot completely preserve the freshness of produces [3]. In recent years, active packaging materials have become increasingly prevalent in food supply chains due to their antibacterial and antioxidant properties [4,5]. The use of nanoparticles with unique physicochemical properties and biosafety to improve the performance of films has become a research focus.

Polyvinyl alcohol (PVA) is a petroleum-based polymer with excellent film-forming properties [6]. It is extensively used in film production, healthcare applications, and food packaging [7]. However, a lower water resistance and a higher light transmittance of PVA film limits its application in food fields [8]. Some researchers have found that PVA can be combined with polysaccharides and proteins to prepare composite films with better physicochemical properties than single-component films. For example, the film prepared with PVA and soy protein isolate (SPI) showed excellent mechanical properties [9]. SPI has the characteristics of high thermal stability [10], good biocompatibility, excellent biodegradability, and sustainability [11]. SPI films usually have low mechanical properties and water sensitivity [12]; thus, they require mixing or cross-linking modification with other materials. It was reported that the interaction of SPI and PVA improved the mechanical strength [13] and film-forming ability of pure SPI films [14], enhancing the degradability and thermal stability of pure PVA films [15]. However, SPI/PVA composite films showed a higher water absorption than single-component films [16], restricting their use in the field of food packaging.

Carbon dots (CDs) are nanomaterials smaller than 10 nm. CDs have the advantages of excellent water solubility [17], biocompatibility [18], low cost, stable fluorescent properties [19], excellent antimicrobial [20], and antioxidant activities [21]. Currently, they have been employed as nanofillers in packaging films made from biopolymers. The presence of a large number of -COOH, -NH_2_, and -OH on the surface of CDs makes it easy to form hydrogen bonds with other polymers, making the composite film tougher [22,23].

This study pioneers the incorporation of CDs as multifunctional nanofillers into an SPI/PVA dual-polymer matrix, transcending the constraints of traditional single-component nanoparticle modifications to achieve concurrent enhancement in both functional properties and physical-barrier performance. The composite films were thoroughly assessed for their morphological features, crystallinity, barrier performance, thermal stability, and bioactive properties. The packaging efficacy was validated through storage tests on green jujubes, meatballs, and soybean oil samples, confirming its practical value in perishable food preservation.

## 2. Materials and Methods

### 2.1. Materials

Bananas, green jujubes, soybean oil, pork back fat, and beef were bought from the Yonghui Store in Yangzhou, China. PVA powder (1799, alcoholysis degree of 98–99%) and SPI powder (protein content > 90%) were purchased from Jiangsu Nanjing Bast Biological Co., Ltd. (Nanjing, China). All other chemicals were purchased from Jiangsu Nanjing Bast Biological Co., Ltd. *E. coli* and *S. aureus* were acquired from the Institute of Microbiology, Chinese Academy of Sciences (Beijing, China).

### 2.2. Fabrication of CDs

The CDs were produced using the hydrothermal process described by Mehta et al. [24], with minor adjustments. Briefly, 100 mL of 30% ethanol (*v*/*v*) and 30 g of peeled banana paste were blended, and the blend was then transferred to the hydrothermal reactor. The mixture underwent a six-hour heating reaction at a temperature of 180 °C inside an oven. The reaction products were allowed to naturally cool to ambient temperature before being centrifuged at 5000 rpm for 20 min. The supernatant after centrifugation was filtered by 0.22 μm membrane. The CD powder was then freeze-dried after 500 Da dialysis for 48 h. The fabricated CDs were characterized by transmission electron microscopy (TEM), X-ray pattern, Raman, Fourier-transfer infrared (FTIR), fluorescence, and UV-Vis spectroscopy [25].

#### 2.2.1. Antibacterial Activity of CDs

##### Minimum Inhibitory Concentration (MIC) and Growth Curve

The antibacterial property of CDs was evaluated using the MIC method. The CD powder was dissolved and diluted with the sterile liquid medium to create concentrations of 100, 50, 25, 12.5, 6.25, and 3.125 mg/mL. Then, 100 μL of CDs with different concentrations was mixed with 100 μL of indicator bacteria (*E. coli* and *S. aureus*, about 7 log10 CFU/mL) in a microtiter plate. At the same time, 100 μL of indicator bacteria and 100 μL of sterile liquid medium were mixed as the control. In addition, to guarantee the sterility of the liquid medium, a blank control consisting of 200 μL of the liquid medium was established. Bacteria were cultured at 37 °C for 24 h, and the turbidity of each pore in the microporous plate was observed. The smallest concentration of CDs corresponding to the wells that did not show turbidity was the MIC of CDs [26]. Meanwhile, the absorbance at 600 nm of the samples at different periods (0, 3, 6, 9, 12, 24, 36, and 48 h) was determined, and the growth curves were plotted.

##### Integrity of Cell Membrane

The cell integrity of bacteria was examined according to the method described by Lin et al. [27], with slight modifications. After activation, the bacteria (*E. coli* and *S. aureus*) were washed 2~3 times and resuspended in 0.1 mol/L phosphate buffer solution (PBS, pH = 7.4). Bacterial suspensions were added with different concentrations of CDs (0, 1, 2, 3, and 4 mg/mL) and incubated at 37 °C for 8 h. The supernatant was obtained by centrifugation at 8000 rpm for 5 min. The absorbance at 260 nm was expressed. In addition, the protein content was determined by using the Caumas Brilliant Blue method.

##### Permeability of Cell Wall

To determine the effect of CDs on cell wall permeability, alkaline phosphatase (AKP) leakage was detected in the liquid supernatant after 0, 1, 2, 3, and 4 mg/mL CD treatment [27]. The activity of AKP was determined using an AKP assay kit produced by Nanjing Jianjian Bioengineering Institute of Nanjing, China.

#### 2.2.2. Antioxidant Activity of CDs

The antioxidant activity of CDs was assessed using the ABTS radical scavenging assay [28]. ABTS radical solution (7.4 mM ABTS with 2.6 mM K_2_S_2_O_8_) was incubated in darkness for 12 h and adjusted to A₇₃₄ = 0.7 ± 0.02 with 95% ethanol. Reaction mixtures containing 1.6 mL ABTS solution and 0.4 mL CDs (0.0625~1 mg/mL) were incubated for 30 min under dark conditions. Absorbance measurements (A_0_, sample; A_i_, ABTS control) were performed at 734 nm. A blank group was set to remove the interference of CDs. The scavenging rate was calculated as follows:

(1) $$\mathrm{ABTS}\;\mathrm{scavenging}\;\mathrm{rate}\;(\%)=\frac{A_{0}-A_{i}}{A_{0}}\;\times \;100\;$$

Moreover, DPPH radical scavenging activity of CDs was evaluated following the method of Jamróz et al. [29], with some modifications. DPPH solution (0.01 g in 250 mL 95% ethanol) was mixed with CD solutions (1:1 *v*/*v*) and incubated in darkness for 30 min. Absorbance measurements at 517 nm included experimental groups (A_i_: DPPH + CDs) and controls (A_0_: DPPH alone). A blank group was set to remove the interference of CDs. Scavenging activity was calculated as follows:

(2) $$\mathrm{DPPH}\;\mathrm{scavenging}\;\mathrm{rate}\;(\%)=\frac{A_{0}-A_{i}}{A_{0}}\;\times \;100\;$$

### 2.3. Preparation of Composite Film

PVA solution: 5 g of PVA was dissolved in 100 mL of distilled water (30 min hydration) and then stirred in 95 °C water for 1 h. SPI solution: 5 g of SPI was dispersed in 100 mL of distilled water under magnetic stirring for 1 h and then filtered through double-layered gauze. The SPI and PVA solutions were blended at a 1:5 mass ratio, followed by the addition of CDs to achieve final concentrations of 0.0, 0.4, 0.6, 0.8, and 1.0% (*w*/*w*). Glycerol (20 wt% of total dry mass) was incorporated as a plasticizer. The mixture (15 mL) was cast into 10 cm × 10 cm petri dishes and dried for 48 h in a fume hood to produce composite films designated as SPI/PVA and x% CD/SPI/PVA (x = 0.4, 0.6, 0.8, and 1.0).

#### 2.3.1. Characterization of Films

The FTIR spectra of films were obtained using an FTIR spectrometer (FTIR, Cary 610/670, Varian, Palo Alto, CA, USA) with a spectral range for scanning of 400–4000 cm^−1^. The XRD pattern of the films was analyzed using an X-ray diffractometer (XRD, D8 Advance, Bruker AXS, Karlsruhe, Germany), using the scanning angle at 5–50° and a scanning speed of 6°/min. The thermal stability of films was studied by the thermogravimetric analyzer (Pyris 1 TGA, PerkinElmer, Waltham, MA, USA), using the temperature range of 30–700 °C, with nitrogen (N_2_) gas as the shielding gas.

#### 2.3.2. Properties of Films

##### Light Transmittance

The light transmittance of films at 280 nm (T_280_) and at 660 nm (T_660_) was measured using a UV-Vis spectrophotometer (Lambda 35, PerkinElmer, Waltham, MA, USA).

##### Water Vapor Transmission Rate (WVTR) and Water Vapor Permeability (WVP)

The WVTR and WVP of films was measured according to Huang et al. [30], with some modifications. Films were sealed onto weighing bottles (50 mm × 50 mm) containing 3 g of anhydrous CaCl_2_, using petroleum jelly. The bottle was weighed with the film attached. Subsequently, it was introduced into a dryer holding 1000 mL of distilled water at 25 °C (100% RH). After 2 h of equilibration, the vials were removed and weighed at regular intervals, with no more than a 5% increment in mass at the end of the weighing. The calculation methods of WVTR and WVP were as follows:

(3) $$\mathrm{WVP}=\frac{{∆m\;}^{*}\;d}{{A\;}^{*\;}{∆t\;}^{*\;}∆p}$$

(4) $$\mathrm{WVTR}=\frac{∆m}{{A\;}^{*}\;∆t}$$

where Δm is the mass increment (g), d is the thickness of the film (m), A is the area of the film (m^2^), Δt is the measurement time interval (s), and Δp is the water vapor pressure difference (Pa).

##### Water Contact Angle (WCA)

Film samples (10 mm × 60 mm) were attached to slides with double-sided adhesive. Then, 10 μL of distilled water was dropped onto the membrane surface using a micro syringe, and the contact angle was immediately measured using a WCA meter (ZJ-7000, Zhi Jia Instruments, Shenzhen, China). Three copies were made, and each sample was measured six times.

##### Water Absorption Rate

The water absorption rate of films was measured according Maciel et al. [31], with made some modifications. Films were cut into 50 mm × 50 mm squares, dried at 50 ± 2 °C for 24 h (initial mass m_1_), and then immersed in distilled water (25 °C) for 24 h. Surface moisture was absorbed with filter paper, and the film samples were weighted (m_2_) within 2 min. Water absorption rate (%) was calculated using the following:

(5) $$\mathrm{Water}\;\mathrm{absorption}\;(\%)=\frac{m_{2\;}-m_{1}}{m_{1}}\;\times \;100\;$$

##### Antioxidant Activity

The ABTS radical scavenging activity of films was measured according to Li, Lin, Gao, Han, and Chen [28], with some modifications. First, 4 mL of ABTS analytical solution was mixed with 0.1 g of chopped film. The absorbance of ABTS solution containing film (A_i_) and ABTS solution (A_0_) at 734 nm was measured after 30 min reaction under dark conditions. A blank group was set to remove the interference of CDs. The ABTS clearance rate was calculated as Equation (1).

For DPPH analysis, the technique outlined by Jamróz, Kopel, Tkaczewska, Dordevic, Jancikova, Kulawik, Milosavljevic, Dolezelikova, Smerkova, and Svec [29], with a few modifications, was implemented. A 0.1 mM of DPPH ethanol solution was prepared. The cut film (0.1 g) was mixed with DPPH solution (4 mL). The absorbance of DPPH solution-containing films (A_i_) and DPPH solution alone (A_0_) at 517 nm was measured after a 30 min reaction under dark conditions. A blank group was set to remove the interference of CDs. The clearance rate was calculated as Equation (2).

##### Antibacterial Activity

Using the attachment method, the inhibitory efficacy of films against *E. coli* and *S. aureus* was examined. The test bacteria (7 log10 CFU/mL) were inoculated onto the surface of agar plate using a sterilized coating rod. The film (1 cm × 1 cm) was positioned horizontally on the surface and left to incubate at a temperature of 37 °C for the entire night. The area where the film made contact with the agar was examined for the formation of colonies.

In addition, the changes in the growth curves of the *E. coli* and *S. aureus* under different film solutions were tested. Test groups contained 100 μL film solution mixed with 100 μL bacterial inoculum (7 log10 CFU/mL), while controls received 100 μL inoculum + 100 μL sterile medium. Sterility controls (200 μL medium alone) were included. Plates were incubated at 37 °C for 24 h with OD600, with monitoring at defined intervals to generate growth curves.

##### Release Kinetics

As per the methodology proposed by Das et al. [32], the release rates of the CDs were investigated by food stimulants. Square film samples (2 cm × 2 cm) were placed in 20 mL of different food-simulating solutions (water, 4% acetic acid, and 65% ethanol solution for simulating water-containing, acidic, and alcoholic foods, respectively) and oscillated at 25 °C. After specific time intervals (0, 0.5, 1, 1.5, 2, 2.5, and 3 h), 200 μL of individual solution was taken, and the absorbance at 395 nm was measured.

### 2.4. Packaging Test

To evaluate the preservation efficacy of CD/SPI/PVA composite films, storage experiments were systematically conducted on green jujube and meatball samples. Results indicated that the 0.8% CD/SPI/PVA film exhibited optimal performance in preservation activity and functionality, establishing it as an optimal packaging material. In contrast, the 1.0% formulation was deemed unsuitable for packaging applications due to inferior optical properties (dark coloration and low light transmittance).

#### 2.4.1. Green Jujube Storage Test

Fresh green jujubes (intact, disease-free, uniform ripeness, and without mechanical damage) were selected, washed with deionized water to remove surface contaminants, and divided into three groups. Two experimental groups were packaged with SPI/PVA and 0.8% CD/SPI/PVA films, while a third group remained unpackaged, serving as the control. All samples were stored under ambient temperature conditions (25 ± 2 °C) for 9 days to simulate real-world storage. Surface-color parameters (L*, a*, and b*) were measured every 3 days, using a colorimeter (CR-410, Konica Minolta, Tokyo, Japan), and weight loss rate was quantified at identical intervals. The calculation formula was as follows:

(6) $$\mathrm{Weightlessness}=\frac{A_{2}-A_{1}}{A_{1}}\;\times \;100\%$$

where A_1_ is the original mass, and A_2_ is the measured mass.

#### 2.4.2. Meatball Storage Test

The meatballs were formulated with beef (66%), pork back fat (14%), water (14%), and auxiliary ingredients (6%). Fresh beef and fat were diced, homogenized with auxiliary ingredients, and shaped into 50 ± 2 g meatballs. The prepared meatballs were cooked in boiling water (100 °C, 15 min), cooled to ambient temperature (25 ± 2 °C, 40 min), and then packed. Three experimental groups were established: two packaged with SPI/PVA or 0.8% CD/SPI/PVA films, and an unpackaged control group. All samples underwent refrigerated storage (4 ± 0.5 °C) for 14 days. Then, changes in total bacterial count (TBC), pH, and thiobarbituric acid reactive substance (TBARS) of freshly prepared samples (FP sample) and refrigerated samples were determined.

The TBC of meatballs was measured according to Yuan et al. [33], with some modifications, and the result was expressed in log CFU/g.

The pH value was determined using a pH meter. A 10 g homogenized sample was mixed with 100 mL deionized water, homogenized, and filtered, and the filtrate pH was measured in triplicate.

The TBARS was determined following the method proposed by Wang et al. [34]. The calculation formula was as follows:

(7) $$TBARS/(mgMDA/kg)=\frac{A_{532}-A_{600}}{155}\times \frac{1}{10}\times 72.6\times 100$$

where A_532_ is the absorbance at 532 nm, and A_600_ is the absorbance at 600 nm.

#### 2.4.3. Oil Storage Test

The PV of soybean oil was measured according to Rakariyatham et al. [35], with some modifications. Small glass bottles containing 3 mL soybean oil were sealed with PVA film, SPI/PVA film, and 0.8% CD/SPI/PVA film, respectively. These glass bottles were then transferred to a desiccator. After 20 days of storage, the peroxide value (PV) of the soybean oil in the bottles was determined.

### 2.5. Statistical Analysis

The performance of the films was measured using three separate films as a repeated experimental unit. Statistical evaluation was conducted through one-way ANOVA with Duncan’s multiple range test (α = 0.05), using IBM SPSS Statistics (v.27). Origin 2021 was used for mapping figures.

## 3. Results and Discussion

### 3.1. Morphology of CDs

The morphology of the fabricated CDs was examined using TEM. As displayed in Supplementary Figure S1, the CDs had an approximately spherical shape and were evenly distributed without any clumping.

### 3.2. Characterization of Films

#### 3.2.1. FTIR Analysis

**Figure 1:**
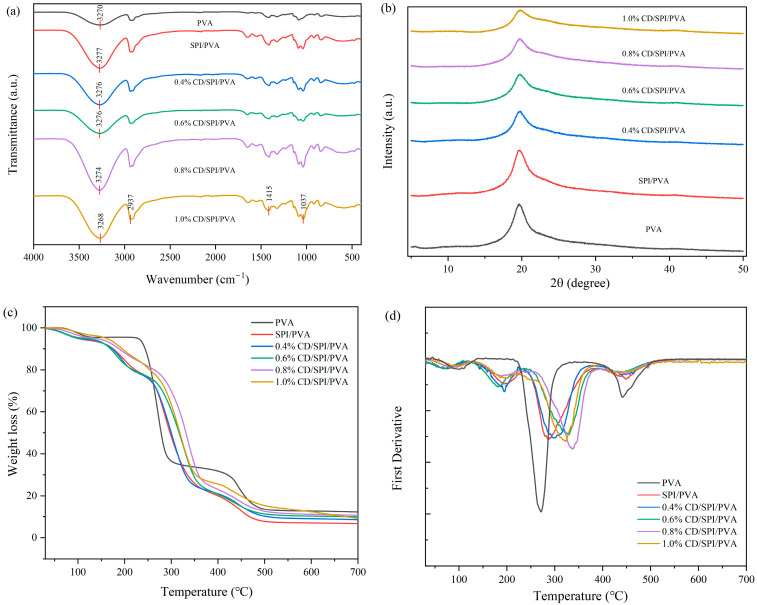
(**a**) FTIR spectrum, (**b**) XRD patterns, (**c**) weight loss, and (**d**) first derivative of different films.

Figure 1a displays the FTIR spectra of the films. The band at 2937 cm^−1^ was caused by the tensile vibration of the C-H bond [30]. The band observed at around 1415 cm^−1^ was the bending vibration of the C-H bond. The tensile vibration near 1037 cm^−1^ was attributed to the C-O bond [22]. The apparent band observed in films containing CDs were comparable to that in SPI/PVA film, with no significant changes, except that the intensity of the band varied slightly. The results indicated that CDs had high compatibility with polymer matrix. The O-H stretching vibrations in SPI/PVA film were observed at a band of 3277 cm^−1^. In 0.4%-, 0.6%-, 0.8%-, and 1.0%-CD/SPI/PVA films, the band slightly shifted to lower wave numbers of 3276, 3276, 3274, and 3268 cm^−1^, respectively. This shift indicated the occurrence of numerous hydrogen bonds between CDs and the SPI/PVA polymer matrix.

#### 3.2.2. XRD Analysis

The impact of CDs on the crystalline phase of the polymer matrix was examined using XRD analysis. The findings are displayed in Figure 1b. The diffraction peak of PVA film at 2θ was 19.6°, reflecting its crystalline structure [36]. By comparison, the diffraction peak was almost unchanged by the addition of SPI. No new diffraction peaks were produced by the addition of CDs, possibly due to the addition of CDs at very small concentrations. The diffraction peaks of the CD/SPI/PVA films correspond to the (101) crystal plane of PVA, reflecting the crystallographic properties of its monoclinic crystal system.

#### 3.2.3. Thermogravimetric Analysis

Thermal stability is of utmost importance for all materials for their applications in packaging fields, where the materials must be sterilized at around 100~121 °C [37]. Figure 1c displays the weight loss of the films with various concentrations of CDs, whereas Figure 1d presents the results of the first-order derivative. All the composite films showed almost the same multi-step thermal decomposition spectrum. The films were quite stable at 110~138 °C. The initial thermal degradation process took place between temperatures of 30~150 °C, which might be caused by the volatilization of water and glycerol. When the temperature exceeded 150 °C, the second thermal degradation began, which might be caused by the heat breakdown of SPI and PVA. Thermal decomposition of the biopolymer backbone was mainly at 150~230 °C. The thermal decomposition of PVA was dominated at 250~350 °C; however, the curve of composite film exhibited a noticeable upward shift as the concentration of CDs increased within this temperature range. At 275 °C, the mass loss of pure SPI/PVA or 0.4% CD/SPI/PVA films was similar, which was about 32.8%. The weight loss rates of 0.6% CD/SPI/PVA and 0.8% CD/SPI/PVA films gradually decreased to 27.4% and 21.9%, respectively, while the weight loss rate of 1.0% CD/SPI/PVA was only 24.7%. The third thermal degradation occurred at 350~465 °C; the films containing CDs exhibited a greater mass loss compared to the pure PVA films, possibly due to the deterioration of the carbon matrix structure. The findings demonstrated that including CDs inside the films improved their thermal stability within the temperature range of 250~350 °C. The thermal stability of 1.0% CD/SPI/PVA was weaker than that of 0.8% CD/SPI/PVA film, which might be ascribed to an excessive incorporation of CDs. Moreover, the heat resistance temperature of the films containing CDs was as high as 110~138 °C, making the film heat-resistant during sterilization and therefore suitable for packaging applications.

### 3.3. Film Properties

#### 3.3.1. Light Transmittance

**Figure 2:**
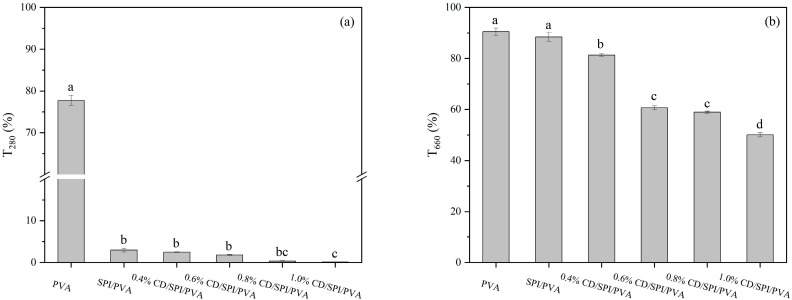
Transmittance of different films at (**a**) 280 nm and (**b**) 660 nm. Note: Different groups with different lowercase letters mean significant difference (*p* < 0.05).

The transmittance at 280 nm (T280) and 660 nm (T660) wavelengths was measured to evaluate the UV protection and transparency of the film. Figure 2a,b illustrate the results. The T280 and T660 of PVA films were 77.8% and 90.5%, respectively. After the addition of SPI, the T280 of films experienced a notable drop, suggesting a substantial improvement in the UV protection of films (*p* < 0.05). Normally, SPI contains aromatic amino acids, such as tyrosine and phenylalanine, which possess benzene rings in their side-chain structure, enabling them to absorb UV light [38]. The T660 of SPI/PVA film was slightly reduced, and the biggest reason might be that the SPI solution was an emulsion, which resulted in reduced film transparency. The T280 of CD/SPI/PVA films exhibited a substantial drop (*p* < 0.05) with the increase in CD concentration. The UV-light absorption properties of CDs might be responsible for this phenomenon. The UV protection of the packaging film helps to prevent the discoloration and oxidation of food products. For 1.0% CD/SPI/PVA, the UV-blocking effect was the strongest, with almost zero UV transmittance; however, the transmittance of visible light decreased dramatically, from 88.4% to 50%, affecting the transparency of films. The materials used for packaging need to maintain a high level of transparency. In addition, the addition of CDs makes the film darker. With the increase in CD concentration, the total color difference (Δ*E*) of the composite film was significantly increased (Supplementary Table S1).

#### 3.3.2. WVP, WVTR, WCA, and Water Absorption Rate

**Figure 3:**
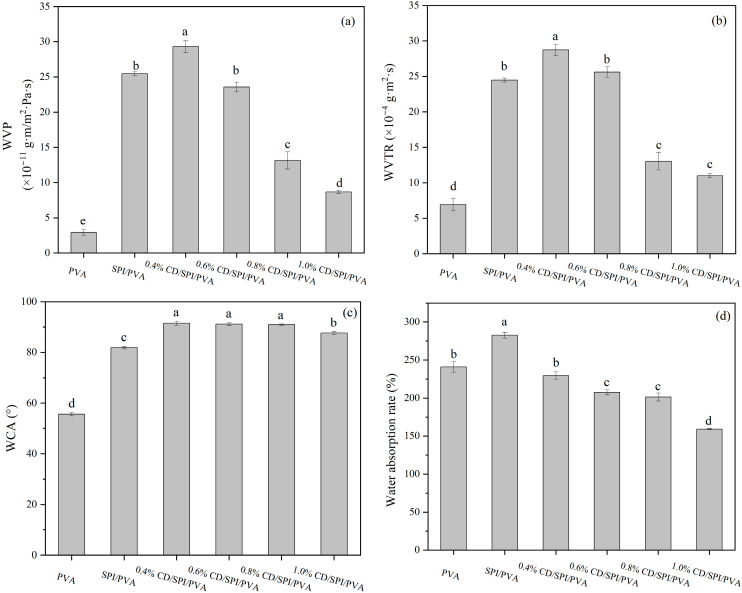
(**a**) Water vapor permeability, (**b**) water vapor transmission rate, (**c**) water contact angle, and (**d**) water absorption rate of different films. Note: Different groups with different lowercase letters mean significant differences (*p* < 0.05).

Figure 3a,b display the WVP and WVTR of films. The WVTR of PVA film was measured to be 6.9 × 10^−4^ g·m^2^·s, while the WVP was found to be 2.9 × 10^−11^ g·m/m^2^·s·Pa. The use of SPI resulted in a significant increase in WVP or WVTR (*p* < 0.05). PVA is a macromolecule polymer, giving it a better barrier property. However, the addition of SPI might have disturbed the interior of molecular regular arrangement and hydrogen bonding network, leading to the appearance of more pore structures, which increased water vapor transmission.

The WVP and WVTR of 0.4% CD/SPI/PVA film were higher than that of SPI/PVA film, which might be attributed to an unequal dispersion of few CDs inside the polymer matrix, forming tiny channels [39]. However, as the concentration of CDs continued to increase, such tiny channels might be sufficiently blocked, increasing the internal densification of the film and thus enhancing its water vapor-barrier properties. The SEM images in Supplementary Figure S2 support this hypothesis. The CD/SPI/PVA composite films showed a denser cross-section compared to the SPI/PVA film. The inclusion of CDs resulted in a substantial decrease in WVP (*p* < 0.05), and the WVP value of 1.0% CD/SPI/PVA film was the lowest. Although CDs have good hydrophilicity, the homogeneous dispersion of CDs inside the polymer matrix prevents the diffusion of water vapor [40]. Enhancing the vapor resistance of the film is beneficial for extending the duration that food can be stored [41].

The surface wettability of films was systematically characterized through WCA analysis. As shown in Figure 3c, The PVA film exhibited inherent hydrophilicity (WCA < 65°), consistent with its hydroxyl-rich polymer matrix [42]. The addition of SPI induced substantial hydrophobicity enhancement (WCA = 81.83°). Subsequent CD loading synergistically amplified this effect: 0.4%, 0.6%, and 0.8% CD/SPI/PVA film achieved a hydrophobic surface (WCA = 91.48°, 91.14°, and 90.88°). Notably, excessive CD content reversed this trend, and the WCA of 1.0% CD/SPI/PVA film decreased to 87.64°, which may be related to the hydrophilicity of CDs.

Figure 3d illustrates the water absorption rate of the films. The PVA film exhibited a water absorption rate of 240.9%. Actually, PVA has good water-absorbing properties because its molecule contains a significant quantity of polar hydroxyl groups (-OH), making it capable of establishing robust hydrogen-bonding connections with water molecules [43]. The water absorption rate of pure SPI/PVA film was 289.9%, while the use of SPI substantially enhanced the value (*p* < 0.05). The porous structure inside the SPI/PVA film leads to easier penetration of water molecules into the film, making the composite film highly absorbent, as evidenced by SEM results. The water absorption rates of CD/SPI/PVA films were significantly lower than those of SPI/PVA films (*p* < 0.05). The addition of CDs might enhance the interaction force between molecules inside the film, leading to an enhanced film densification and reduced pore formation, thus reducing the adsorption and permeation of water molecules by the film. Food packaging materials with a high water-absorption rate undergo water-absorption swelling, increasing the possibility of microbial reproduction. Therefore, the packaging materials should be designed to minimize water absorption.

### 3.4. Antioxidant Activity of CDs and Films

**Figure 4:**
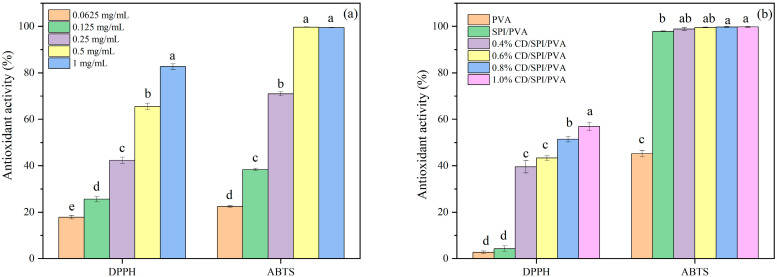
DPPH and ABTS radical scavenging activities of (**a**) CDs and (**b**) films. Note: Different groups with different lowercase letters mean significant difference (*p* < 0.05).

Figure 4a displays the DPPH and ABTS radical scavenging activities of CDs. With the increase in CD concentration, the clearance rate of ABTS rose from 22.5% to 99.5%, while the clearance rate of DPPH rose from 17.9% to 82.7%. Due to the presence of several organic functional groups on the surface of CDs (such as hydroxyl, carboxyl, and amino groups), the transfer of H· (a free radical) to ABTS-H and DPPH-H was facilitated [40]. The clearance rate of CDs to ABTS was higher than that of DPPH. Since the hydrophilic CDs are more dispersed and stabilized in ABTS solution prepared with water, they expose more reaction sites, showing a better interaction with free radicals.

Using packaging-film materials that possess antioxidant properties can effectively prolong the freshness of the product [37]. Therefore, the antioxidant activity of the films was assessed. The results are displayed in Figure 4b. The antioxidant activity of PVA film was weak, with a clearance rate of 2.69% for DPPH and 45.24% for ABTS. The ABTS free radical scavenging rate of SPI/PVA film was significantly higher than that of PVA due to the existence of flavonoid compounds in SPI [44], which was able to introduce more molecules of hydroxyl groups, enhancing the antioxidant capacity of composite film. With the addition of CDs, the antioxidant activity of the composite films was further enhanced. When the addition amount was 1.0%, the scavenging rate of DPPH and ABTS reached 56.93% and 99.80%, respectively. The difference in scavenging rate between DPPH and ABTS is related to the hydrophilicity of PVA, making the composite film easy to decompose in ABTS aqueous solution and, thus, leading to a faster release rate of CDs than in DPPH ethanol solution [2].

### 3.5. Antibacterial Properties of CDs and Films

#### 3.5.1. Antibacterial Activity of CDs

**Figure 5:**
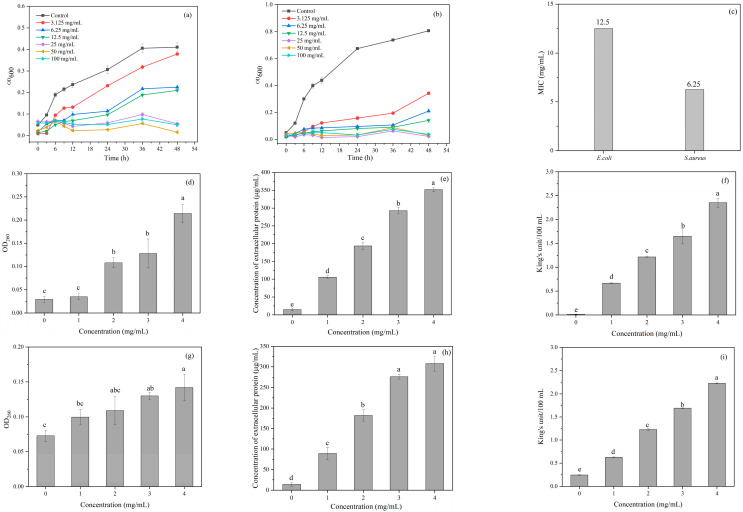
Growth curves of (**a**) *E. coli* and (**b**) *S. aureus* under different CDs concentrations; (**c**) MIC values for *E. coli* and *S. aureus*; and (**d**) nucleic acid leakage, (**e**) protein leakage, and (**f**) AKP activity of *E. coli* under different CDs concentrations. (**g**) Nucleic acid leakage, (**h**) protein leakage, and (**i**) AKP activity of *S. aureus* under different CDs concentrations. Note: Different groups with different lowercase letters mean significant difference (*p* < 0.05).

The antibacterial efficacy of CDs was systematically evaluated through MIC analysis and bacterial growth-curve determination. As shown in Figure 5c, CDs demonstrated strain-dependent antimicrobial activity, exhibiting a lower MIC against Gram-positive (*S. aureus*, 6.25 mg/mL) compared to Gram-negative (*E. coli*, 12.5 mg/mL). This selectivity correlates with structural differences in bacterial cell walls, as Gram-negative bacteria possess an additional outer membrane that hinders antimicrobial penetration. Dose-dependent relationships were confirmed through real-time monitoring of bacterial growth kinetics (OD600, Figure 5a,b), where increasing CDs concentrations (0–100 mg/mL) progressively inhibited microbial proliferation in both strains.

#### 3.5.2. Inhibitory Mechanism of CDs

##### Integrity of Cell Membrane

To investigate the membrane-disruptive mechanism of CDs, we quantified cytoplasmic component leakage through OD260 (nucleic acids) and protein-release measurements (Figure 5d,e,g,h). Both *E. coli* and *S. aureus* exhibited dose-dependent leakage profiles when exposed to CDs (0–4 mg/mL). At 4 mg/mL CDs, the OD260 of *E. coli* increased from 0.029 to 0.214, and the OD260 of *S. aureus* increased from 0.073 to 0.142 compared with the control group. Both bacteria also showed a significant increase in extracellular protein concentrations, similar to the trend of nucleic acid leakage. The results indicated that CDs caused irreparable damage to the cell membrane, leading to the leakage of intracellular components and ultimately to cell death.

##### Permeability of Cell Wall

AKP is a hydrolase localized between the cell membrane and wall that is often used to reflect the breakage of the cell wall. As shown in Figure 5f,i, AKP levels in the bacterial suspension increased with the increasing CD concentration after 8 h of treatment. At CD concentrations of 4 mg/mL, the AKP levels of *E. coli* and *S. aureus* were 2.35 and 2.23 King’s unit/100 mL, respectively, which were significantly higher than those in the control (*p* > 0.05). This is consistent with the findings that the disruption of cell wall permeability leads to bacterial death and increased extracellular AKP activity [27]. The results suggest that CDs can disrupt the cell wall of *E. coli* and *S. aureus*, leading to increased cell membrane permeability and increased AKP leakage.

#### 3.5.3. Antibacterial Activity of Films

**Figure 6:**
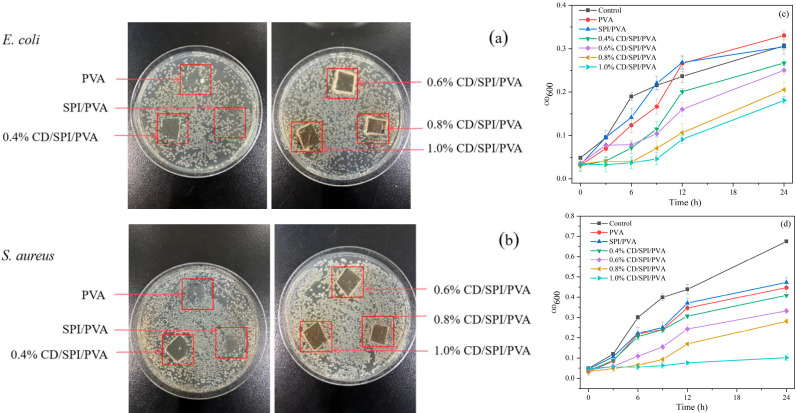
Antimicrobial activity of different films against (**a**) *E. coli* and (**b**) *S. aureus*. The growth curve of (**c**) *E. coli* and (**d**) *S. aureus* treated with different films.

The antibacterial efficacy of the films was assessed using the film adhesion method and bacterial growth curve method. As shown in Figure 6a,b, there were obvious colonies of *S. aureus* and *E. coli* under PVA film and SPI/PVA film, but no colony growth was observed under CD/SPI/PVA composite films, demonstrating that the composite film containing CDs had significant bacteriostatic activity. Because the CDs are only quantum in size, they can penetrate the cell walls of microorganisms and disrupt metabolic processes, ultimately leading to the breakdown of the cell [37]. As mentioned above, CDs achieve antibacterial activity by disrupting cell wall integrity and cell membrane permeability. As the CD concentration increased, the area covered by the film gradually turned yellow. This discoloration was a result of the CDs’ release from the film matrix into agar, thus achieving the inhibition effect. However, the diffusion and mobility of CDs in agar were low; thus, it did not produce an inhibition zone around it [41]. Figure 6c,d show the growth curve of *E. coli* and *S. aureus* treated with different films. The composite film containing CDs effectively inhibited the growth of *E. coli* and *S. aureus* within 24 h, with enhanced efficacy against *S. aureus*. With the increase in CDs addition, the antibacterial activity of the composite film against these two bacteria was also significantly enhanced.

### 3.6. Release Kinetics

**Figure 7:**
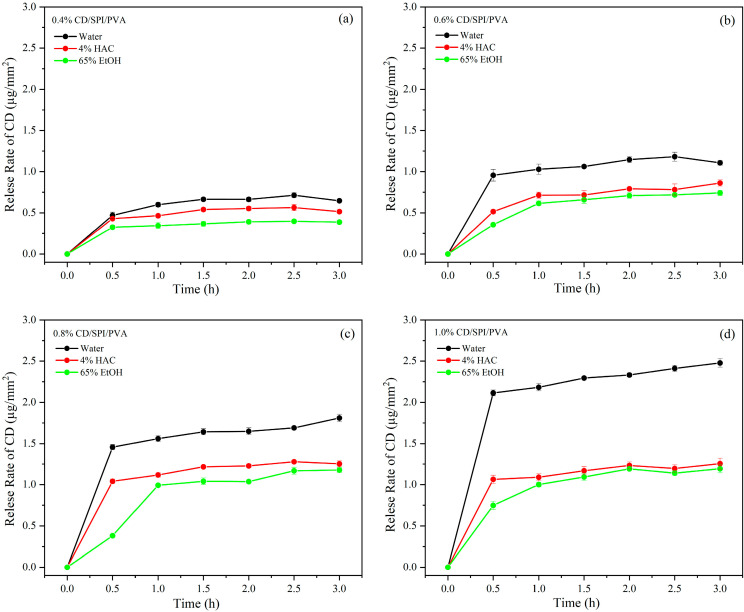
Release rates of CDs from (**a**) 0.4% CD/SPI/PVA, (**b**) 0.6% CD/SPI/PVA, (**c**) 0.8% CD/SPI/PVA and (**d**) 1.0% CD/SPI/PVA films in different food simulants.

The release rate of CDs from films into different simulated liquids is shown in Figure 7a–d. In all three solutions, the release rate of CDs was higher during the first 30 min; it then increased slowly and smoothed out. As the CD concentration increased, the release rate also increased. The release rate in 4% acetic acid and 65% ethanol was lower, compared to that in water. The primary factor contributing to this phenomenon was the aqueous solubility of CDs. Additionally, the solubility of the polymer matrix in different solvents and the affinity of solvents to CDs might affect the release rate [45]. Because the SPI/PVA matrix had a higher water absorption rate, water might easily permeate into the film and thoroughly hydrate it, helping CDs to be released from the film. Acetic acid and ethanol solutions might not be able to effectively interact with the SPI/PVA matrix, thereby preventing the migration and diffusion of CDs in the film. These findings suggested that the type of analog exposure and the concentration of CDs in the film influenced the release rate of CDs. Moreover, a hydrophilic environment led to a higher release rate of CDs, enhancing the biological activity of the film upon contact with food surfaces.

### 3.7. Effect of Active Film on Food Sample Storage

#### 3.7.1. Green Jujubes

**Figure 8:**
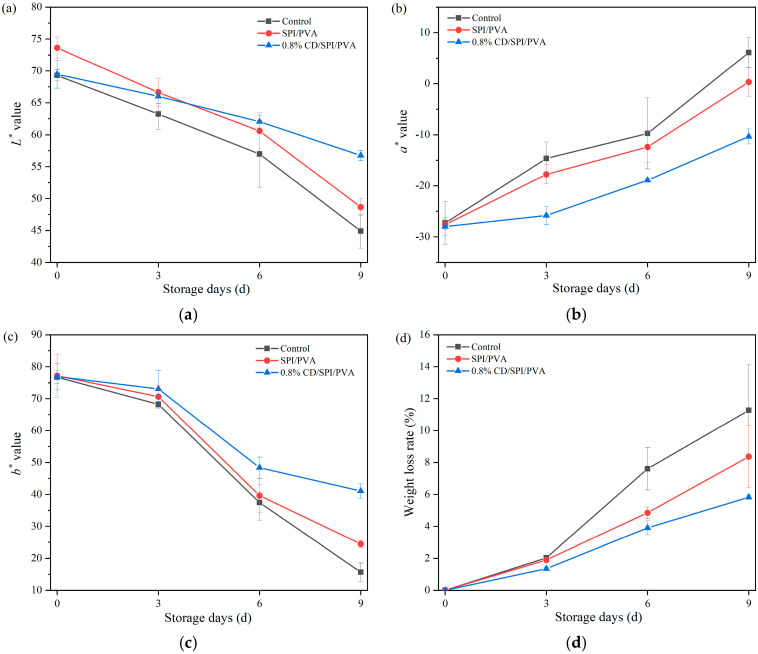
Effect of different packaging films on color parameters: (**a**) L*, (**b**) a*, and (**c**) b*. (**d**) Weight loss rate of green jujube during storage.

Color is an important indicator for evaluating the freshness of fruits. By evaluating the color changes in green jujube during storage, the antioxidant and UV protection properties of CD/SPI/PVA composite film can be reflected. The L*, a*, and b* values represent the attributes of “lightness”, “green/redness”, and “blue/yellowness”, respectively. As shown in Figure 8a–c, the L* and b* values of all groups showed a declining trend. The L* value reflected the change in brightness; the lower the L* value, the darker the color [46]. On the 9th day, the L* values of the control group and SPI/PVA group decreased by 24.83 and 24.96, respectively, which were significantly higher than those of 0.8% CD/SPI/PVA group (*p* < 0.05). The a* value gradually increased over time, suggesting that the green color of jujube gradually faded and became brown. The a* values of the control group and SPI/PVA group exceeded 0 on the 9th day, while the a* value of the 0.8% CD/SPI/PVA group was −10.34, indicating that the CD/SPI/PVA film effectively delayed the oxidative browning of jujube. This may be due to the antioxidant activity of the composite film inhibiting the activity of polyphenol oxidase, preventing it from inducing a browning reaction.

As shown in Figure 8d, the weight loss rate of green jujube in each group showed an upward trend with the extension of storage time. The 0.8% CD/SPI/PVA groups showed the lowest weight loss during the same storage time. This was due to the low water vapor permeability of films, which inhibited the water exchange between jujube and the outside world. At the end of storage, the weight loss rate of the 0.8% CD/SPI/PVA group was 5.82%, which was significantly lower than those of the control group (11.27%) and SPI/PVA group (8.36%) (*p* < 0.05). The results showed that CD/SPI/PVA film could effectively reduce water loss [47], inhibit the respiration and transpiration of jujube, and prolong the storage period.

#### 3.7.2. Meatballs

**Figure 9:**
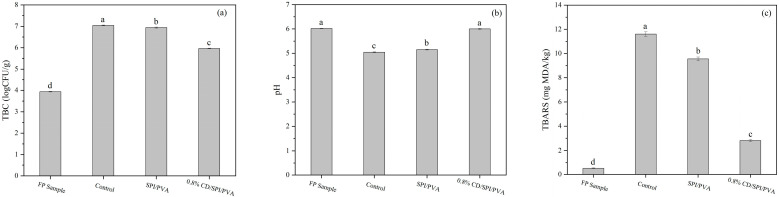
Effect of different packaging films on (**a**) TBC, (**b**) pH, and (**c**) TBARS of refrigerated meatballs. Note: Different groups with different lowercase letters mean significant difference (*p* < 0.05).

The changes in TBC of differently packaged meatballs after 2-week refrigeration at 4 °C are shown in Figure 9a. Compared with the FP sample, the control group (unwrapped) exhibited a significantly higher TBC value (*p* < 0.05). Notably, the 0.8% CD/SPI/PVA group maintained significantly lower TBC levels than both the control and SPI/PVA groups (*p* < 0.05), indicating effective microbial growth suppression by the active CD/SPI/PVA film during storage.

Meat products typically undergo protein and lipid oxidation during storage. The products of both oxidation reactions could promote the decrease in pH value and lead to the quality deterioration of meat products. As shown in Figure 9b, the control group demonstrated the most pronounced pH reduction compared to the FP sample, while the 0.8% CD/SPI/PVA group showed minimal pH changes. Similarly, after 2-week refrigeration at 4 °C, the control group exhibited the highest TBARS value (11.60 mg MDA/kg), which was significantly higher than that of FP sample (0.52 mg MDA/kg, *p* < 0.05). The 0.8% CD/SPI/PVA group maintained a TBARS value of only 2.80 mg MDA/kg (Figure 9c), confirming that the CD-incorporated CD/SPI/PVA composite film effectively alleviated the oxidative rancidity and prolonged the shelf life of the meatballs.

#### 3.7.3. Soybean Oil

**Figure 10:**
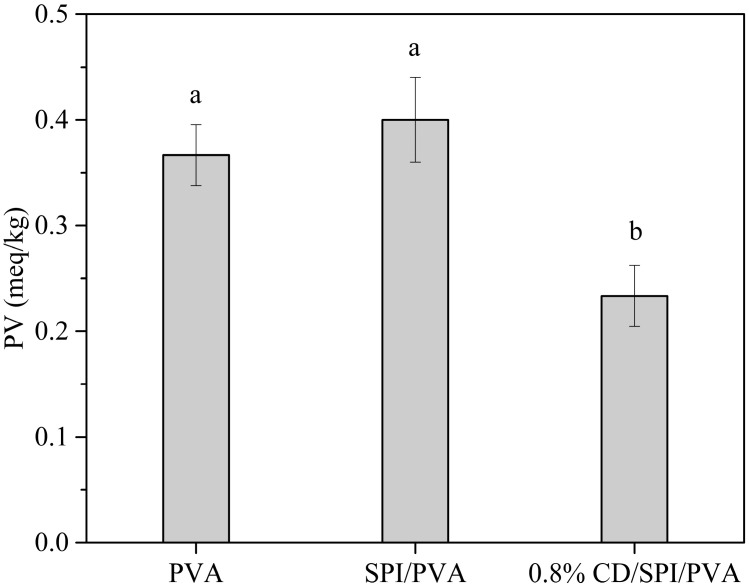
Effect of different packaging films on PV of soybean oils. Note: Different groups with different lowercase letters mean significant differences (*p* < 0.05).

PV is an important indicator to evaluate lipid oxidation. As shown in Figure 10, the PV of 0.8% CD/SPI/PVA film was significantly lower than that of PVA film and SPI/PVA film (*p* < 0.05). This may be due to the fact that the oxygen barrier capacity of 0.8% CD/SPI/PVA film was better than that of PVA film and SPI/PVA film, which delayed the increase in PV. Oxygen is the main factor leading to lipid oxidation, and the insufficient oxygen barrier capacity of the packaging film will accelerate lipid oxidation, thereby increasing the peroxide value. These results further indicate that CD/SPI/PVA composite film has an excellent barrier property, which is beneficial to prolonging the storage period of soybean oil and is suitable for the packaging and storage of fatty foods.

## 4. Conclusions

This study demonstrates the successful development of CDs-reinforced SPI/PVA composite films through a dual-polymer matrix strategy, overcoming the inherent limitations of single-component nanoparticle modifications. The integration of CDs conferred synergistic improvements in both functional properties and physical-barrier performance. Comprehensive characterization validated enhanced thermal stability and sustained bioactive release kinetics. Practical storage trials on perishable foods demonstrated remarkable efficacy: green jujubes exhibited a 48% lower weight loss rate, meatballs showed 76% lower TBARS values, and soybean oil showed a 36% lower PV. These findings establish a scalable approach for designing next-generation active food packaging systems with dual functionality, addressing critical challenges in shelf-life extension and quality preservation.

## Data Availability

Data is contained within the article or Supplementary Materials.

## Supplementary Materials

The following supporting information can be downloaded at: https://www.mdpi.com/article/10.3390/antiox14060669/s1, Figure S1: TEM image of CDs; Figure S2: (a) Digital images, (b) surface, and (c) cross-sectional SEM images of different films; Table S1: The surface color parameters of the films; Table S2: Mechanical properties of the films. References [48,49,50,51] are cited in the supplementary materials.
