# Supplementary material for: Carbon Dots-Enhanced Soy Protein Isolate/Polyvinyl Alcohol Composite Film for Active Preservation of Oxidation-Sensitive Foods
Source: Antioxidants (Basel). 2025 May 31;14(6):669. doi: 10.3390/antiox14060669 (PMC12189048; doi:10.3390/antiox14060669)
Supplement: Supplementary file 1 [file antioxidants-14-00669-s001.zip › antioxidants-3636653-supplementary.pdf]

Supplementary Materials

Carbon Dots-Enhanced Soy Protein Isolate/Polyvinyl Alcohol Composite Film for Active Preservation of Oxidation-Sensitive Foods

Linlin Zhao [1,2], Huinan Jiang [1,2,3], Zhengxuan Han [1,2], Wenqin Gu [1,2], Bimal Chitrakar [4] and Xiangren Meng [1,2,5,*]

1 College of Tourism and Culinary Science, Yangzhou University, Yangzhou 225127, China; 008113@yzu.edu.cn (L.Z.); mz120232117@stu.yzu.edu.cn (H.J.); 212402502@stu.yzu.edu.cn (Z.H.); mx120241311@stu.yzu.edu.cn (W.G.)
2 Key Laboratory of Chinese Cuisine Intangible Cultural Heritage Technology Inheritance, Ministry of Culture and Tourism, Yangzhou 225127, China
3 College of Food Science and Engineering, Yangzhou University, Yangzhou 225127, China
4 College of Food Science and Technology, Hebei Agricultural University, Baoding 071001, China; bimal@hebau.edu.cn
5 Chinese Cuisine Promotion and Research Base, Yangzhou 225127, China
* Correspondence: xrmeng@yzu.edu.cn

S1. Materials and methods

S1.1 Characterization of CDs and films

The fabricated CDs have been characterized by transmission electron microscopy (TEM). The morphology of films was studied by scanning electron microscope (GeminiSEM 300, Carl Zeiss GMBH, UK). The digital images of films was captured bu camera function of the phone.

S1.2 Properties of films

S1.2.1 Color parameters

Film color parameters were measured using a CR-410 colorimeter following Min, *et al.* [1]. Using a standard white panel as the reference, $L*$, $a*$ and $b*$ values were determined, representing the attributes of "lightness", "green/redness" and "blue/yellowness", respectively. Total color difference (ΔE) was calculated as:

$$\Delta E = \sqrt{\Delta L^{*2} + \Delta a^{*2} + \Delta b^{*2}} \quad (1)$$

where, the differences in color values between the film and white panel were represented by $\Delta L*$, $\Delta a*$ and $\Delta b*$, respectively.

S1.2.2 Mechanical properties

The film samples were cut into strips (1 cm × 6 cm) and the thickness was measured using a digital display electronic caliper. The mechanical properties of the films, such as tensile strength (TS), elongation at break (EB), and elastic modulus (EM) were measured using a texture analyzer. The fixture used probe A/TG, the fixture spacing was 40 mm, and the speed before testing was 1 mm/sec, the running speed was 1 mm/sec, and the speed after running was 10 mm/sec.

S2. Results and discussion

S2.1 Characterization of CDs and films

The morphology of the fabricated CDs was examined using TEM. As displayed in Fig. S1, the CDs had an approximately spherical shape and were evenly distributed without any clumping.

The digital images of films are displayed in Fig. S2a. The PVA films and SPI/PVA films were highly transparent and colorless, while the CD/SPI/PVA films with added CDs had a mild yellow hue. The color intensified as the CDs level increased and the transparency gradually decreased.

SEM photography was used to capture the cross-sectional and surface morphologies of films. The outcomes are displayed in Fig. S2b - S2c. The surface of pure SPI/PVA film exhibited a consistently smooth texture, without any cracks or defects, which suggested a high level of compatibility between two biopolymers. The improved compatibility of SPI might be due to the inclusion of glycerol, a water-soluble substance utilized as a plasticizer [2]. Glycerol has hydrogen bonds to make SPI and PVA more compatible [3]. The introduction of CDs did not result any substantial alteration to the surface morphology of films, showing smooth surface. However, a porous structure appeared inside SPI/PVA film as shown in the cross-sectional images. The steady decrease in the porosity structure within the composite film was seen with the addition of CDs, which made the compactness of the film's interior composition. Attributed to an appropriate size of CDs and many organic functional groups on its

surface, the CDs hydrogen-bonded with the SPI/PVA polymer matrix [4] to form a denser network structure, which was also confirmed by the FTIR findings (Next section). The SEM results showed that the CDs had good compatibility with the SPI/PVA polymer matrix.

S2.2 Color parameters of films

The color parameters of PVA film and composite films are shown in Table S1. PVA film has the highest brightness, which indicates that PVA mainly contributed to the brightness of the composite film [1]. The $L*$ value gradually decreased after the addition of SPI. The addition of CDs makes the film darker. With the increase of CDs concentration, $L*$ value decreased significantly, indicating that the overall brightness of the film decreased and the color darkened. However, the $a*$ value of the composite film was increased, and the change trend of the $b*$ value increased first and then slightly decreased, but the overall trend was increasing, which was caused by the brown CDs. As a result, the total color difference (ΔE) of the composite film was significantly increased.

S2.3 Mechanical properties

The thickness and mechanical properties of the films are shown in Table S2. The addition of SPI significantly increased the thickness of the composite film compared with the PVA film. However, due to the homogeneous dispersion of small-sized CDs in the polymer matrix, the incorporation of CDs had no significant effect on the thickness

of the composite films ($p > 0.05$). The SPI/PVA film had better mechanical properties with TS of 17.12 MPa, EB of 393.10%, and EM of 4.35 GPa.The addition of CDs decreased the TS and EB of the composite film. Similar to the results of this study, it has been reported that the incorporation of CDs into gel/poly(vinyl alcohol)-based films resulted in significant reduction in TS and EB, while no significant difference in EM of the composite films [1].

References

1. Min, S.; Ezati, P.; Yoon, K.S.; Rhim, J.-W. Gelatin/poly (vinyl alcohol)-based functional films integrated with spent coffee ground-derived carbon dots and grapefruit seed extract for active packaging application. *International Journal of Biological Macromolecules* **2023**, *231*, 123493, doi:10.1016/j.ijbiomac.2023.123493.
2. Krishnaveni, T.; Ramasubbu, A. Synthesis and characterization of biomimetic hydroxy apatite-silver impregnated soy protein isolate nanocomposites for dental implantations. *Asian J. Chem* **2017**, *29*, 2634-2638, doi:10.14233/ajchem.2017.20755.
3. Su, J.-F. Biodegradable soy protein isolate/poly (vinyl alcohol) packaging films. *Handbook of Composites from Renewable Materials* **2017**, *30*, 587, doi:10.1002/9781119441632.ch103.
4. Min, S.; Ezati, P.; Rhim, J.-W. Gelatin-based packaging material incorporated with potato skins carbon dots as functional filler. *Industrial Crops and Products* **2022**, *181*, 114820, doi:10.1016/j.indcrop.2022.114820.

Table S1. The surface color parameters of the films

Films	L^*	a^*	b^*	ΔE
PVA	87.05 ± 0.76^{a}	1.70 ± 0.10^{e}	-4.12 ± 0.01^{e}	13.70 ± 0.70^{e}
SPI/PVA	86.34 ± 0.60^{a}	1.15 ± 0.04^{e}	-1.67 ± 0.10^{d}	13.81 ± 0.60^{e}
0.4%/CD/SPI/PVA	70.35 ± 1.05^{b}	4.98 ± 0.43^{d}	25.72 ± 0.59^{c}	39.57 ± 1.22^{d}
0.6%/CD/SPI/PVA	62.89 ± 0.47^{c}	8.89 ± 0.49^{c}	29.98 ± 0.71^{a}	48.52 ± 0.87^{c}
0.8%/CD/SPI/PVA	52.80 ± 1.29^{d}	11.86 ± 1.01^{b}	28.32 ± 1.13^{ab}	53.88 ± 1.23^{b}
1.0%/CD/SPI/PVA	50.38 ± 1.17^{d}	14.29 ± 0.63^{a}	26.51 ± 0.80^{bc}	58.05 ± 0.80^{a}

Note: Different letters within a column represent significant difference ($p < 0.05$)

Table S2. Mechanical properties of the films

Films	Thickness (μm)	TS (MPa)	EB (%)	EM (GPa)
PVA	42.33 ± 6.35^{b}	34.33 ± 0.50^{a}	215.53 ± 8.63^{e}	15.94 ± 0.41^{a}
SPI/PVA	104.33 ± 5.86^{a}	17.12 ± 0.95^{b}	393.10 ± 15.51^{a}	4.35 ± 0.07^{b}
0.4%/CD/SPI/PVA	102.00 ± 16.00^{a}	9.56 ± 0.61^{c}	301.47 ± 5.60^{cd}	3.17 ± 0.16^{b}
0.6%/CD/SPI/PVA	92.00 ± 1.73^{a}	12.67 ± 0.83^{c}	345.40 ± 7.14^{b}	3.67 ± 0.31^{b}
0.8%/CD/SPI/PVA	101.33 ± 13.01^{a}	12.33 ± 1.63^{c}	328.44 ± 18.50^{bc}	3.75 ± 0.49^{b}
1.0%/CD/SPI/PVA	79.33 ± 12.74^{a}	8.90 ± 2.57^{c}	284.95 ± 4.30^{d}	3.13 ± 0.92^{b}

Note: Different letters within a column represent significant difference ($p < 0.05$)

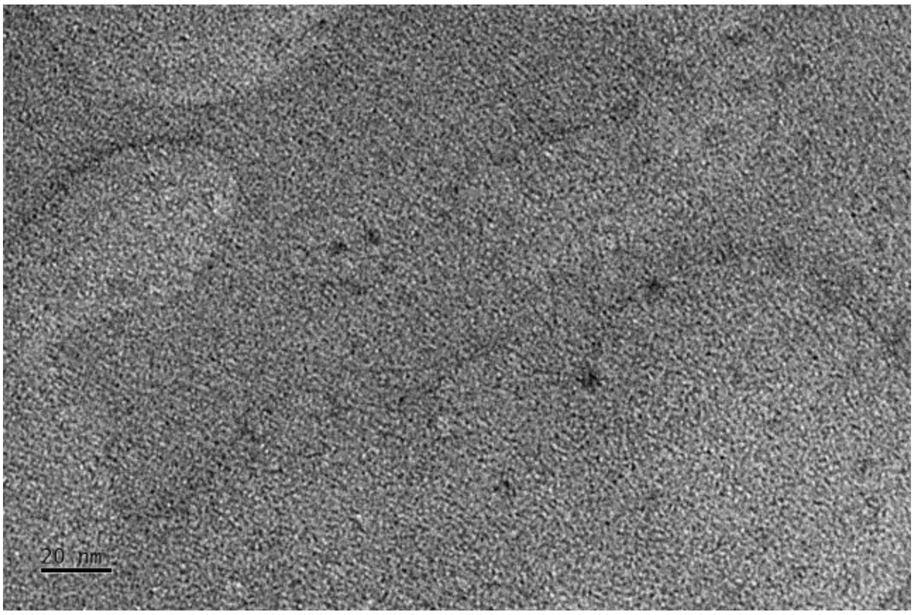

Figure S1. TEM image of CDs.

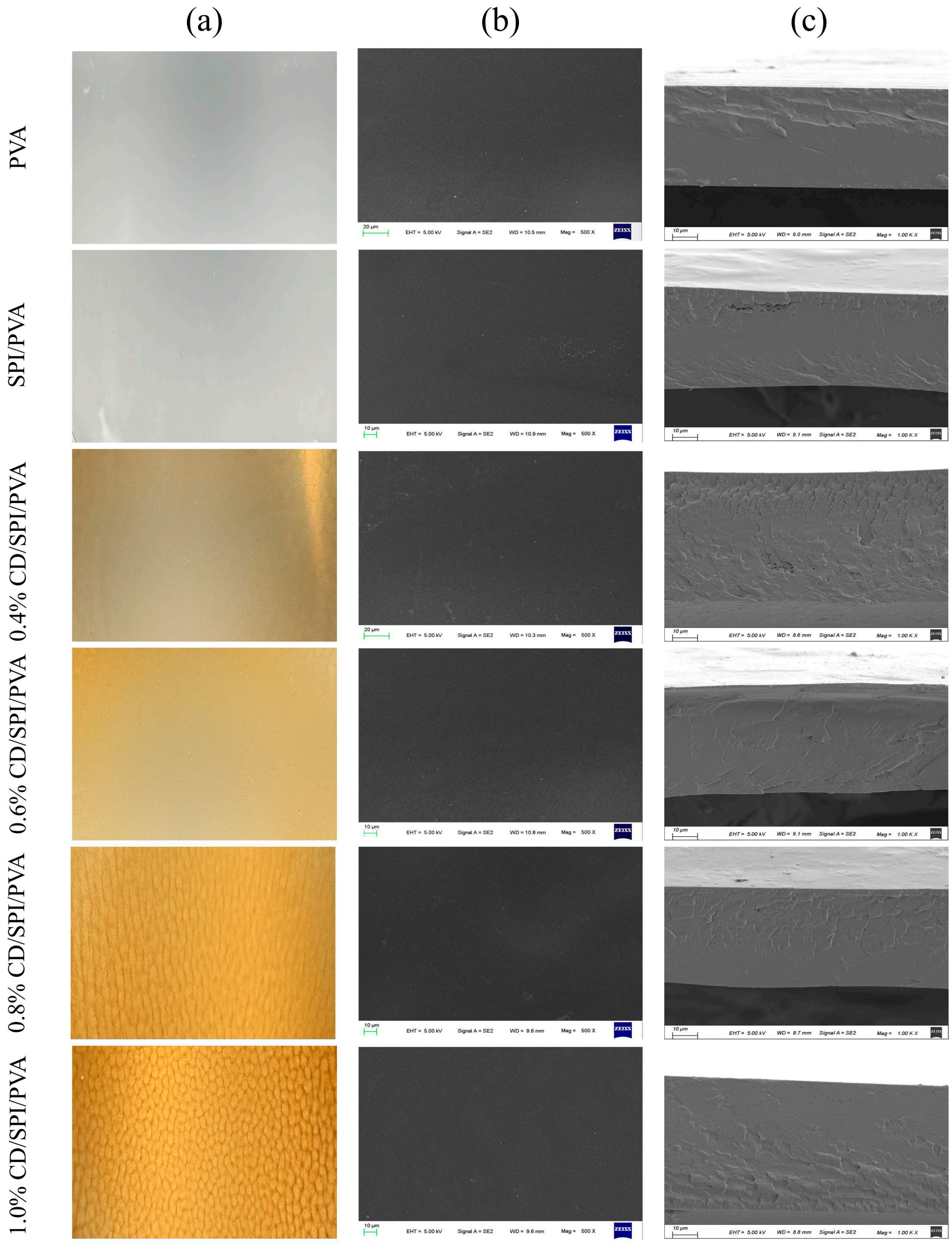

Figure S2. (a) Digital images, (b) surface, and (c) cross-sectional SEM images of different films.
